# Prevalence, risk factors and treatment outcomes of syphilis among people living with human immunodeficiency virus at primary care clinics in Malaysia: A retrospective study

**DOI:** 10.51866/oa.471

**Published:** 2024-02-07

**Authors:** Abdul Hafiz Gani, Narul Aida Saleh, Sheela Bai Selvam, Iskandar Azwa

**Affiliations:** 1 MBBS, MFamMed, MRCGP, MSc, CMID, Klinik Kesihatan Mahmoodiah, Jalan Mahmoodiah, Johor Bahru, Johor, Malaysia. Email: hafizgani@yahoo.com; 2 MBBS, MFamMed, Klinik Kesihatan Kuala Lumpur, Kuala Lumpur, Malaysia.; 3 MBBS, MFamMed, Klinik Kesihatan Cheras, Jalan Yaacob Latiff, Kuala Lumpur, Malaysia.; 4 MBChB, FRCP, Dip GUM, Dip HIV, Faculty of Medicine, University of Malaya, Kuala Lumpur, Malaysia.

**Keywords:** Syphilis, HIV, Primary care, Malaysia

## Abstract

**Introduction:**

Syphilis and human immunodeficiency virus (HIV) coinfection is a common clinical problem with a significant rising trend worldwide. In Malaysia, the burden of care is shared between hospitals and primary care clinics. This study aimed to determine the prevalence of syphilis among people living with HIV (PLHIV) at primary care clinics in Malaysia and assess its association with sociodemographic characteristics, risk factors and treatment outcomes.

**Methods:**

This retrospective study included 750 PLHIV aged ≥18 years who attended primary care clinics in three different locations in Malaysia from 1 January 2017 to 31 December 2019. Data were obtained from the patients’ clinical notes using a structured questionnaire evaluating the sociodemographic characteristics, history of sexual and lifestyle behaviours, diagnosis and management.

**Results:**

The patients’ age ranged from 18 to 78 years (mean=34.7, standard deviation=10.2). The prevalence of syphilis among the PLHIV at the three primary care clinics was 33.8% (n=254). Syphilis was significantly associated with gender (P=0.038) as well as sexual activity (P<0.001), substance use (P=0.038), history of chemsex (P=0.001) and history of sexually transmitted infections (STIs) (P<0.001) within the past 12 months. The majority of the PLHIV with syphilis received treatment at the primary care clinics (n=248, 97.3%), and up to 96.1% (n=245) had completed such treatment.

**Conclusion:**

Syphilis is prevalent among PLHIV at primary care clinics, and most patients receive standard treatment. Therefore, primary care doctors must enhance their knowledge to effectively manage STIs, especially syphilis.

## Introduction

Human immunodeficiency virus (HIV) infection and syphilis are significant sexually transmitted infections (STIs) worldwide. The increasing incidence of syphilis at an early stage among people living with HIV (PLHIV) is worrying because it can facilitate the transmission of HIV and increase the incidence of new HIV infection later.^1^ Coinfection with syphilis and HIV is predictable because of similar risk factors. Both diseases are synergistic in their transmission process, making the diagnosis and treatment more complex.^2^

The prevalence of syphilis among PLHIV differs across countries. In Thailand, 16.1% of PLHIV are coinfected with syphilis and have higher HIV ribonucleic acid levels in their blood, seminal and rectal secretions than those with no syphilis.^3^ In Singapore, the incidence of syphilis increased 20 times from 2010 to 2017 and was consistently higher among men who have sex with men (MSM) than among heterosexual men.^4^ A 5-year retrospective study on PLHIV with syphilis among MSM at several hospitals in Malaysia found that latent syphilis was the most common stage of diagnosis, and the failure rate was about 8.5% after a year of treatment.^5^

There are several possible risk factors of syphilis, including insufficient immune defensive mechanism against HIV regardless of antiretroviral therapy (ART) status, risk compensation in sexual behaviour and susceptibility to Treponema pallidum infection.^6^ The recurrence of syphilis is associated with previous syphilis episodes, sexual risk behaviour, history of condomless anal sex with casual sexual partners and ART.^7^ The risk behaviours that contribute to infection among patients with STI and HIV in Malaysia include low condom use, increased number of sexual partners, history of STIs and history of sex with foreigners.^8^

This study aimed to assess the prevalence, risk factors and treatment outcomes of syphilis among MSM with HIV at selected STI primary care clinics in Malaysia. To date, there are no available published data on the prevalence, treatment and outcomes of syphilis among PLHIV at the primary care level in Malaysia.

## Methods

This retrospective study included PLHIV who attended three primary care clinics in Malaysia, including the Mahmoodiah Primary Care Clinic in Johor Bahru and the Kuala Lumpur Primary Care Clinic and the Cheras Primary Care Clinic in Kuala Lumpur. Most newly diagnosed PLHIV on first-line ART receive care at a public primary care clinic in Malaysia. The three clinics were selected based on the largest number of PLHIV in public primary care. The patients were selected based on the registration number from January 2017 to December 2019, and those who met the following inclusion criteria were included: age of ≥18 years, diagnosis of HIV, registration from 1 January 2017 to 31 December 2019 and maintenance of activity for at least a year after registration.

The sample size of 750 participants was calculated using the Kish formula. The estimated prevalence of PLHIV in Malaysia was 115,263,9 indicating that a total of 662 participants were required. With a 99% confidence level, a 0.05 significance level and an 80% power, each clinic needed to collect 250 participants to achieve a total of 750 participants. The participants were randomly selected using standardised steps generated using an online randomiser and based on the registration number.

Data on sociodemographic characteristics and risk factors and management of syphilis were collected using data collection forms that were filled in manually by three investigators at the different study sites. This study collected information on sociodemographic characteristics, risk factors, sexual history, treatment status, treatment outcome and notification and status of the partner(s) of patients diagnosed with syphilis. Syphilis was diagnosed based on laboratory findings (positive rapid plasma regain (RPR) with a confirmatory Treponema pallidum partial agglutination (TPPA)).

The data were analysed using the Statistical Package for the Social Sciences (SPSS) version 26 (IBM SPSS Statistics, Armonk, NY, USA). Continuous variables were presented as means and standard deviations and categorical variables as numbers and percentages. The association between syphilis and the studied variables was assessed for statistical significance using the chi-square test or Fisher’s exact test, as appropriate. Multiple logistic regression (MLR) analysis was conducted to identify the factors associated with syphilis. A P-value of <0.05 was considered statistically significant.

Ethical approval for this study was obtained from the National Medical Research Register, Malaysia (approval number: NMRR-20-3101-57889 [IIR]; research ID: 57889).

## Results

A total of 750 participants from each primary care clinic were included, with 250 participants randomly selected from each clinic. The mean age of the participants was 34.7±10.2 years, and the largest proportion was aged 18–29 years. The sociodemographic characteristics and risk factors of the patients are shown in Table 1. The majority of the PLHIV were homosexual (n=441, 58.8%), and up to 52.5% were sexually active within the past 12 months. Table 2 shows the diagnosis and stage of syphilis among the PLHIV. The prevalence of syphilis among the PLHIV was 33.9% (n=254), and the most common stage of syphilis was late latent syphilis (68.5%, n=174).

**Table 1:** Sociodemographic characteristics and risk factors.

Variable		n	%
**Age: 18–78 years (mean=34.7, SD=10.2)**			
Age group (year)	18-29	281	37.5
	30-39	276	36.8
	40-49	121	16.1
	50-59	50	6.7
	≥60	22	2.9
Ethnicity	Malay	427	56.9
	Chinese	221	29.5
	Indian	63	8.4
	Bumiputera	23	3.1
	Foreigner	7	0.9
	Others	9	1.2
Gender	Man	684	91.2
	Woman	48	6.4
	Transwoman	18	2.4
Marital status	Single	546	72.8
	Married/with partner	171	22.8
	Unknown	33	4.4
Highest educational level	No formal education	6	0.8
	Primary education	33	4.4
	Secondary education	380	50.7
	Tertiary education	238	31.7
	Unknown	93	12.4
Employment status	Employed	575	76.7
	Unemployed	136	18.1
	Unknown	39	5.2
**Risk factor**		**n**	**%**
Sexual orientation	Heterosexual	104	13.8
	Homosexual	441	58.8
	Bisexual	119	15.9
	Unknown	20	2.7
Sexual activity within the past	Yes	394	52.5
12 months	No	274	36.5
	Unknown	207	27.6
Consistency of condom use	Every time	78	10.4
	Most of the time	33	4.4
	About half of the time	32	4.3
	Some of the time	31	4.1
	None of the time	327	43.6
	Unknown	249	33.2
Substance use within the past 12 months	Yes	128	17.1
	No	557	74.3
	Unknown	65	8.7
History of chemsex within the past 12 months	Yes	89	11.9
	No	540	72.0
	Unknown	121	16.1
History of STI within the past 12 months	Yes	89	11.9
	No	540	72.0
	Unknown	121	16.1
Total		750	100

**Table 2:** Diagnosis and stage of syphilis.

Status	Stage	n (%)
Syphilis	Primary	17 (2.3)
	Secondary	38 (5.1)
	Early latent	25 (3.3)
	Late latent	121 (16.1)
	Late latent unknown duration	53 (7.1)
	Tertiary	1 (0.1)
**No syphilis**		496 (66.1)
**Total**		750 (100)

Definition: Primary: Incubation period of 2–3 weeks (range=9–90 days) with local infection, Secondary: Incubation period of 6–12 weeks (range=1–6 months) with generalised infection, Early latent: Asymptomatic syphilis with a duration of <2 years, Late latent: Asymptomatic syphilis with a duration of ≥2 years, Late latent unknown duration: Asymptomatic syphilis with an unknown duration, Tertiary: Late symptomatic syphilis including syphilitic gumma, cardiovascular syphilis and/or neurosyphilis.

Table 3 shows the logistic regression analysis of the factors associated with syphilis among the PLHIV. In the simple logistic regression analysis, gender, marital status, educational level, employment status and sexual orientation as well as sexual activity, condom use, substance use, history of chemsex and history of STI within the past 12 months were found to be significant. The MLR analysis revealed that syphilis was significantly associated with gender, sexual activity, substance use, history of chemsex and history of STI.

**Table 3:** Simple and multiple logistic regression analyses of the factors associated with the risk of syphilis.

Variable	**Status of syphilis**		**Logistic regression analysis**			
	Negative (n=495) n (%)	Positive (n=255) n (%)	Simple		Multiple	
			OR (95% Cl)	P-value	OR (95% Cl)	P-value
**Age group (year)**						
18-29	172 (61.2)	109 (38.8)	13.3(1.8-100.4)	0.004	3.6 (0.4-31.6)	0.249
30-39	178 (64.5)	98 (35.5)	11.6(1.5-87.3)	0.032	3.0 (0.3-26.6)	0.321
40-49	86 (71.1)	35 (28.9)	8.5 (1.1-66.0)	0.506	2.7 (0.3-24.4)	0.377
50-59	38 (76.0)	12 (24.0)	6.6 (0.8-54.6)	0.198	4.3 (0.4-40.8)	0.210
≥60	21 (95.5)	1 (4.5)	1		1	
**Ethnicity**						
Malay	282 (66.0)	145 (34.0)	1		1	
Chinese	144 (65.2)	77 (34.8)	0.7 (0.3-1.7)	0.753	0.8 (0.3-2.1)	0.572
Indian	44 (69.8)	19 (30.2)	0.5 (0.1-3.3)	0.761	0.7 (0.3-2.1)	0.769
Bumiputera	13(56.5)	10(43.5)	0.6 (0.2-1.5)	0.501	1.2 (0.4–3.8)	0.765
Foreigner	5(71.4)	2 (28.6)	0.7 (0.3-1.6)	0.978	0.7 (0.1-6.7)	0.341
Others	7 (77.8)	2 (22.2)	0.4 (0.1-2.2)	0.453	3.1 (0.2-32.7)	0.662
**Gender**						
Man	434 (63.5)	250 (36.5)	1		1	
Woman	47 (97.9)	1 (2.1)	27.0 (3.7-197.4)	<0.001	10.5(1.3-84.7)	0.027
Transwoman	14 (77.8)	4 (22.2)	13.4(1.4-130.1)	0.286	4.3 (0.4-48.8)	0.238
**Marital status**						
Single	341 (62.5)	205 (37.5)	1		1	
Married/with partner	132 (77.2)	39 (22.8)	2.0(1.4-3.0)	0.001	1.8(1.1-2.9)	0.023
Unknown	22 (66.7)	11 (33.3)	1.7 (0.8-3.8)	0.934	1.2 (0.5-3.2)	0.670
**Highest educational level**						
No formal education	4 (66.7)	2 (33.3)	1		1	
Primary education	31 (93.9)	2(6.1)	0.1 (0-1.2)	0.001	0.1 (0-2.0)	0.142
Secondary education	250 (65.8)	130 (34.2)	1.0 (0.2-5.8)	0.902	0.5 (0.1-3.4)	0.440
Tertiary education	151 (63.4)	87 (36.6)	1.2 (0.2-6.4)	0.314	0.4 (0.1-3.0)	0.368
Unknown	59 (63.4)	34 (36.6)	1.2 (0.2-6.6)	0.578	0.5 (0.1-3.8)	0.481
**Employment status**						
Employed	361 (62.8)	214(37.2)	1		1	
Unemployed	108 (79.4)	28 (20.6)	0.4 (0.3-0.7)	<0.001	0.8 (0.4-1.3)	0.312
Unknown	26 (66.7)	13 (33.3)	0.8 (0.4-1.7)	0.928	0.8 (0.3-1.7)	0.517
**Sexual orientation**						
Bisexual	78 (65.5)	41 (34.5)	1		1	
Homosexual	258 (58.5)	183 (41.5)	1.3 (0.9-2.1)	0.165	0.8 (0.4–1.6)	0.562
Heterosexual	143 (84.1)	27 (15.9)	0.4 (0.2-0.6)	<0.001	1.0 (0.6-1.6)	0.939
Unknown	16 (80.0)	4 (20.0)	0.4 (0.1-1.5)	0.180	0.6 (0.2-2.3)	0.464
**Sexual activity within the past 12 months**						
No	225 (82.1)	49 (17.9)	1		1	
Yes	217 (55.1)	177 (44.9)	3.7 (2.6-5.4)	<0.001	3.1 (1.9–5.2)	<0.001
Unknown	53 (64.6)	29 (35.4)	2.5 (1.5–4.3)	0.001	1.2 (0.6-2.5)	0.592
**Condom use within the past 12 months**						
No	234 (71.3)	94 (28.7)	1		1	
Yes	130 (60.5)	85 (39.5)	1.6 (1.1–2.3)	0.009	0.8 (0.5–1.3)	0.324
Unknown	131 (63.3)	76 (36.7)	1.4 (1.0–2.1)	0.052	1.2 (0.7-2.1)	0.468
**Substance use within the past 12 months**						
No	392 (70.4)	165 (29.6)	1		1	
Yes	71 (55.5)	57 (44.5)	1.9 (1.3–2.8)	0.001	2.3 (1.2–4.5)	0.015
Unknown	32 (49.2)	33 (50.8)	2.5 (1.5–4.1)	0.001	0.9 (0.5–1.7)	0.703
**History of chemsex within the past 12 months**						
No	388 (71.9)	152 (28.1)	1		1	
Yes	31 (34.8)	58 (65.2)	4.8 (3.0-7.7)	<0.001	2.8 (1.5-5.5)	0.002
Unknown	76 (62.8)	45 (37.2)	1.5 (1.0-2.3)	0.050	0.6 (0.3-1.1)	0.095
**History of STI within the past 12 months**						
No	328 (76.8)	99 (23.2)	1		1	
Yes	123 (52.8)	110 (47.2)	3.0 (2.1–4.2)	<0.001	2.9 (1.7-5.1)	<0.001
Unknown	44 (48.9)	46 (51.1)	3.5 (2.2-5.5)	<0.001	2.0 (1.4-3.0)	<0.001

n: sample size, OR: odds ratio, P-value: probability value Basic assumptions that must be met for logistic regression include independence of error, linearity in the logit for continuous variables, absence of multicollinearity and lack of strong influential outliers. No multicollinearity was found (VIF=1.085–1.830); Hosmer and Lemeshow test, P>0.05.

Table 4 shows the treatment status and outcome of the PLHIV with syphilis. The majority of the PLHIV with syphilis completed the treatment for syphilis, and most of them responded to such treatment.

**Table 4:** Treatment status and outcome of the PLHIV with syphilis (n=255).

Treatment status	n	%
Treatment complete	245	96.1
Treatment incomplete	6	2.4
Unknown status	4	1.6
Treatment outcome	n	%
Responded to treatment	166	65.1
Treated and defaulted	25	9.8
Reinfected	48	18.8
Failed treatment	16	6.3
Total	255	100.0

Definition: Responded to treatment: Four-fold drop in RPR levels at 1 year, Treated and defaulted: Defaulted after treatment, Reinfection: More than four-fold increase in RPR levels after 6 months of treatment, Failed treatment: No four-fold drop in RPR levels after 6 months and at the end of treatment.

## Discussion

This study found a similar pattern of sociodemographic characteristics among the PLHIV relative to the latest prevalence of HIV transmission in Malaysia.^10^ The majority of the patients with syphilis were aged 18–29 years (n=109, 38.8%), while the minority were aged ≥60 years (n=1, 4.5%). Most patients were single (n=205, 80.4%), and a small number of patients were married/with partners (n=39, 15.3%). These findings are similar to previous reports on syphilis and HIV coinfection.^4,11^

Of the patients in the present study, 85.1% received education at least at the secondary level, and only a small number had no formal education (n=2, 0.8%). These data are different from those of a study conducted in Vietnam, where the total number of patients with syphilis who received primary education and no formal education was up to 19.5%.^12^ Although this study found that employment status was not an independent risk factor of syphilis, a study performed in Brazil suggested that employment is associated with a higher risk of syphilis among PLHIV based on the hypothesis that individuals with stable finances are more likely to engage in high-risk behaviours such as substance use and sex trade, including sex for money or sex for drugs.^13^

The cause and association of syphilis with HIV infection can be related to sexual history, history of STI and lifestyle behaviours, all of which can serve as risk factors. In this study, MSM were the key population, as they made up 87.8% of the individuals positive for syphilis compared with only 10.6% of the heterosexuals. Although this study focused on the general population attending primary care clinics, the majority of those living with HIV in this study were MSM. This proportion may be comparable with that of other studies that have focused on MSM.^4,5,7,11^ Apart from sexual activity, the type of sexual practices and sexual partners are also important factors to explore as risk behaviours. Previous research has connected inconsistent condom use to the transmission of STIs including syphilis, making the advocation of consistently using condoms especially important to PLHIV who are sexually active.^4^

Substance use among the PLHIV in this study showed a slightly less difference between those with and without syphilis. Conversely, a history of chemsex was more common among the PLHIV with syphilis than among those without syphilis. It is known that engaging in chemsex increases the risk of STI, such as having an increased number of partners, and reduces preventive measures, such as infrequent or lack of condom and pre-exposure prophylaxis use.^12^

In the present study, the MLR analysis revealed an association between syphilis and gender after the sociodemographic characteristics, sexual history and lifestyle behaviour were controlled. This finding is similar to most previous reports, where gender is significantly associated with syphilis.^13-16^ Men are known to get involved with activities that can expose them to infection, such as having multiple sexual partners and sex trade.^16^ In this study, the odds ratio of the female patients developing syphilis was 10.5 times higher than that of the male patients, while the odds ratio of the married/with partner patients was 1.8 times higher than that of the single patients. Based on these data, both genders must be considered, and preventive measures must be emphasised for individuals in marital or partnered relationships. Other studies have supported the association of marital status with PLHIV attending specialised care service centres.^8,14^ While it may indicate an association, more focus needs to be given to the lifestyle behaviours of men who are married to reduce the risk of syphilis transmission.

In the MLR model in this study, the independent risk factors of syphilis among the PLHIV included sexual activity within the past 12 months, substance use in a lifetime and chemsex within the past 12 months. The noted association between chemsex and syphilis is similar to the report by DiDomizio within an Asian region.^17^ Conversely, Ahn et al.^18^ reported that there was no indication of reduced syphilis risk with repeated episodes of syphilis. However, the authors considered that this association was limited in evidence because a history of syphilis may not reflect the current disease activity but rather the history of infection (based on the TPPA status). Accordingly, patients may remain positive for TPPA after treatment. However, the association between repeated episodes of syphilis and current active disease based on the TPPA status should be interpreted cautiously owing to the lack of direct testing in the MLR analysis in this study.

The present study found that most PLHIV with syphilis completed the treatment for syphilis (n=245, 96.1%), and most patients responded to such treatment (n=166, 65.1%). This is because most patients in primary care were newly diagnosed and received close monitoring for HIV infection and syphilis. A recent research conducted in a hospital setting in Malaysia revealed that up to 24.7% (n=42) of patients with syphilis were classified to have treatment failure.^19^ This finding is expected at a tertiary centre because most cases are referrals from a primary care centre, where cases can be complex and have been treated previously. Further, the immune system of patients with chronic and advanced stage of HIV infection may encounter challenges in eradicating syphilis.^20^

The limitations of this study include the modest sample size and the cross-sectional design, which may not represent all PLHIV in Malaysia. Nonetheless, the findings are still relevant considering that the majority of PLHIV are attending government outpatient clinics. Other limitations include the incomplete case records and lack of documentation.

Based on the present findings, targeted interventions and preventive measures are recommended for PLHIV in Malaysia. Primary care doctors should educate all PLHIV regardless of gender and marital status on safe sexual practices and the dangers of chemsex to reduce syphilis transmission.

## Conclusion

The prevalence of syphilis among the PLHIV at the three primary care clinics was fairly high (33.8%), with the majority being in the late stage of syphilis (68.5%). Gender, sexual activity, substance use, history of chemsex and history of STI were risk factors of syphilis. A large proportion of the patients (96.1%) completed the treatment for syphilis at the primary care clinics.

## Data sharing statement

The data is only available upon request to the corresponding author.

**How does this paper make a difference in general practice?**

- This study highlights that syphilis is common among people living with HIV (PLHIV).
- Syphilis screening is important among PLHIV for timely treatment.
- Syphilis status should be monitored as part of routine care.
